# Draft Genome Sequence of a Uropathogenic Escherichia coli Sequence Type 44 Strain Carrying Multiple Antimicrobial Resistance Genes

**DOI:** 10.1128/mra.00931-21

**Published:** 2022-03-24

**Authors:** Jessica L. Ortega-Balleza, Abraham Guerrero, Graciela Castro-Escarpulli, Eduardo Cruz-González, Gildardo Rivera, Virgilio Bocanegra-García

**Affiliations:** a Laboratorio Interacción Ambiente-Microorganismo, Centro de Biotecnología Genómica, Instituto Politécnico Nacional, Reynosa, Tamaulipas, Mexico; b Centro de Investigación en Alimentación y Desarrollo, A. C. Unidad Mazatlán, Mazatlán, Sinaloa, Mexico; c Escuela Nacional de Ciencias Biológicas, Instituto Politécnico Nacional, Mexico City, Mexico; University of Maryland School of Medicine

## Abstract

Escherichia coli is a reservoir of antimicrobial resistance genes (ARGs). Here, we report the draft genome sequence of an E. coli strain (31HGR-CBG) that was isolated from a urine sample in Tamaulipas, Mexico. 31HGR-CBG harbors multiple ARGs, including *bla*_CTX-M-15_ and class 1 integron. This strain also carries multiple virulence genes.

## ANNOUNCEMENT

Antimicrobial-resistant Escherichia coli represents a threat to public health (1). Strains of extraintestinal pathogenic E. coli (ExPEC) are genetically diverse and complex (2). ExPEC acts as an opportunistic pathogen, mainly causing urinary tract infections (UTIs) (3). In Mexico, UTIs are a public health problem and represent the third leading cause of morbidity (4, 5).

The 31HGR-CBG strain was isolated in 2018 from a urine sample from a second-level hospital in Reynosa, Tamaulipas, Mexico, from specimens obtained for routine testing of nosocomial pathogens; therefore, neither institutional review board (IRB) approval nor informed consent was required. 31HGR-CBG was grown on Trypticase soy agar and CHROMagar Orientation medium and incubated overnight at 37°C. Standard biochemical tests were also performed.

Genomic DNA was isolated from an overnight culture grown in LB broth (Condalab, Madrid, Spain) at 37°C. DNA was extracted using the Wizard genomic DNA purification kit (Promega, USA). DNA quantification was performed with the Qubit double-stranded DNA (dsDNA) HS assay kit in the Qubit 3.0 fluorometer (Thermo Fisher Scientific, USA). Libraries were constructed with the Nextera Flex library preparation kit and were sequenced using the MiniSeq sequencing system (150-bp paired-end reads). A total of 10,918,316 raw reads were generated. FastQC v0.11.3 (https://www.bioinformatics.babraham.ac.uk/projects/fastqc) and Trim Galore! v0.6.6 (https://www.bioinformatics.babraham.ac.uk/projects/trim_galore) were used to evaluate quality and to trim the raw reads, respectively. Assembly was performed using SPAdes v3.15.2 (6) (with options --isolate and –k 21,31,41,51,61,71,81,91). The quality of the assemblies was assessed with QUAST v5.0.2 (https://github.com/ablab/quast). Contigs smaller than 900 bp were removed. Bacterial identification was confirmed by a BLASTN search (http://blast.ncbi.nlm.nih.gov) against the NCBI database and ribosomal multilocus sequence typing (rMLST) (7).

Automatic annotation was performed by the NCBI Prokaryotic Genome Annotation Pipeline (PGAP) v5.2. Multilocus sequence typing (MLST) was executed with PubMLST v1 (8). Serotype, plasmid replicons, antimicrobial resistance genes (ARGs), and virulence genes were analyzed using SerotypeFinder v2.0 (9), PlasmidFinder v2.1 (10), ResFinder v3.0 (11), and VirulenceFinder v2.0 (12), respectively, available from the Center for Genomic Epidemiology (http://genomicepidemiology.org). ARGs were also predicted with the CARD v3.1.4 database using RGI v5.2.0 (13). Phages were determined by PHASTER (14). Default parameters were used for all software unless otherwise specified.

The genome was 4,981,584 bp in size and was assembled into 141 contigs, with an *N*_50_ value of 91,860 bp, a GC content of 50.82%, and genome coverage of 220×. Annotation identified 4,939 genes, 4,850 coding sequences, and two CRISPR arrays. 31HGR-CBG belongs to sequence type 44 (ST44) and serotype 0101:H4.

Multiple ARGs were identified, including genes conferring resistance to aminoglycosides [*aadA5*, *aph(6)-Id*, *aac(6′)-Ib-cr*, and *aph(3″)-Ib*], extended-spectrum β-lactams (*bla*_CTX-M-15_ and *bla*_OXA-1_), phenicol (*catB3*), macrolides [*mph*(A)], sulfonamides (*sul1* and *sul2*), tetracycline [*tet*(B)], trimethoprim (*dfrA17*), and quaternary ammonium compounds (*qacEΔ1*). In addition, plasmid replicons IncFIA, IncFIB, and IncFII were detected.

Chromosomal mutations in *gyrA*, *parC*, and *parE* were found, indicating fluoroquinolone resistance. Virulence-associated genes and four intact prophages were identified.

### Data availability.

This draft genome has been deposited in GenBank under the accession number JAKJKJ000000000. The reads were deposited in the Sequence Read Archive (SRA) under the accession number SRR15258840.
